# Effect of Aerobic Exposure on Microbial Community Changes and Mycotoxin Content Changes in Corncob Silage

**DOI:** 10.3390/microorganisms14040778

**Published:** 2026-03-30

**Authors:** Xinyi Wang, Xinwen Sun, Dengke Hua, Xinfeng Wang, Wen Shen, Tengyu Wang, Qikai Liu, Xuelian Gao, Yuan Lv

**Affiliations:** College of Animal Science and Technology, Shihezi University, Shihezi 832000, China; 15599814090@163.com (X.W.); wxf-4@163.com (X.W.);

**Keywords:** corncob silage, microbial community, mycotoxins, aerobic exposure

## Abstract

This study aimed to investigate the effects of different aerobic exposure durations on mycotoxin accumulation, nutritional quality changes, and microbial community dynamics of corncob silage. The experiment was divided into four groups: T0 (corncob silage fermented for 45 days without aerobic exposure), T4 (corncob silage exposed to air for 4 days), T8 (corncob silage exposed to air for 8 days), and T12 (corncob silage exposed to air for 12 days). The results showed that after aerobic exposure, the contents of dry matter (DM), crude protein (CP), water-soluble carbohydrates (WSC), Crude Ash, lactic acid (LA), and ammonia nitrogen (NH_3_-N) in all exposed groups (T4, T8, T12) were significantly lower than those in the T0 group, whereas the contents of neutral detergent fiber (NDF), acid detergent fiber (ADF), propionic acid (PA), and butyric acid (BA) were significantly higher than those in the T0 group. Exposure to aerobic conditions for 12 days resulted in the four mycotoxins exhibiting levels significantly higher than those in the other groups, and notably, zearalenone (ZEN) and ochratoxin (OT) exhibited a continuous increase in concentration with the extension of aerobic exposure. Aerobic exposure increased bacterial diversity and fungal relative abundance, and significant separations were observed in both bacterial and fungal communities between the T0 group and the aerobic exposure groups. At the phylum level, *Firmicutes* was the dominant bacterial phylum in the T0 group, while *Pseudomonadota* became the dominant phylum after aerobic exposure. At the genus level, *Lacticaseibacillus* was the dominant bacterial genus in the T0 group, whereas *Variovorax*, *Vibrionimonas*, and *Mycobacterium* dominated the bacterial communities in the aerobic exposure groups. The relative abundance of the fungal phylum *Ascomycota* increased from 30% in the T0 group to 80~90% in the aerobic exposure groups; the dominant fungal genera shifted from Zygosaccharomyces to *Albifimbria* and *Pichia*. In conclusion, prolonged aerobic exposure elevates the concentrations of mycotoxins in corncob silage, reduces the nutritional quality, and induces significant shifts in both bacterial and fungal community compositions.

## 1. Introduction

At present, silage constitutes the primary feed source for ruminants. This ensiling technology not only enhances the nutritional quality of feed but also extends its storage duration and eliminates constraints imposed by ambient climatic conditions [1]. Silage is a feed product fermented anaerobically by lactic acid bacteria naturally occurring on plant tissues, with the advantage of long-term storability. Its core microbial components include lactic acid bacteria, yeasts, enterobacteria, molds, and other bacteria [2]. However, once silage silos or bales are opened, the anaerobic conditions are disrupted, triggering secondary fermentation of the well-fermented silage. This process not only causes dry matter loss but also leads to severe nutrient depletion. Furthermore, aerobic conditions facilitate the proliferation of spoilage microorganisms such as molds, which produce mycotoxins that compromise feeding efficiency [3]. Studies have demonstrated that in whole-plant corn silage contaminated by field molds, the populations of yeasts and molds proliferate more rapidly during aerobic exposure and remain consistently at higher levels [4].

Mycotoxins refer to a class of toxic secondary metabolites synthesized by various mold species. Ingestion of mycotoxin-contaminated feeds by livestock and poultry induces not only a spectrum of toxicological manifestations but also considerable economic losses to the animal production sector, which in turn hinders the advancement of the livestock industry. Notably, these mycotoxins can undergo biomagnification through livestock and poultry, enter the human food supply, and ultimately endanger human health [5]. More than 300 distinct mycotoxins have been identified to date, among which over 40 are amenable to quantitative detection; the common mycotoxins in feed include aflatoxin B1 (AFB1), zearalenone (ZEN), deoxynivalenol (DON) and ochratoxin (OT) [6]. As an effective strategy to mitigate mycotoxin contamination in roughage feeds, ensiling relies on the production of organic acids during anaerobic fermentation to inhibit fungal growth. Organic acids produced during ensiling can inhibit mold growth, with acetic acid content enhancing the aerobic stability of silage. Of note, the storage performance of silage largely depends on its aerobic stability [7].

Aerobic deterioration of silage has long been a research hotspot in the livestock industry; however, most studies have focused on the fermentation process, with relatively limited attention paid to the aerobic exposure phase [8]. This study aimed to investigate the dynamics of mycotoxins, nutritional quality, and microbial community changes during aerobic exposure, thereby providing a theoretical basis for ensuring feed safety and mitigating aerobic spoilage in subsequent applications.

## 2. Materials and Methods

### 2.1. Fermentation Process

Corncobs were collected from Bole Zonghai Jiahui Feed Company (Bole, China) in May 2024 and crushed to a size of 1–3 cm. Then, 0.5% microbial preparation (the microbial preparation used in this study was a specialized silage fermentation inoculum developed by the Feed Comprehensive Utilization Laboratory at Shihezi University, with an initial composition of *Saccharomyces cerevisiae* at 2.5 × 10^8^ CFU/mL, *Bacillus subtilis* at 5.5 × 10^8^ CFU/mL, *Lactobacillus plantarum* at 2.5 × 10^8^ CFU/mL, *Geotrichum candidum* at 7.5 × 10^8^ CFU/mL, and *Candida utilis* at 1.0 × 10^8^ CFU/mL) was evenly sprayed onto the surfaces of corncobs. In addition, 0.2% salt (based on total weight) was added as an energy source for microorganisms, and 200 g·kg^−1^ corn steep liquor (purchased from Xinjiang Wujiaqu Meihua Biological Company) (Wujiaqu, China) was used as the nitrogen source. The samples were evenly mixed until the moisture content reached 65%, with a total weight of 1000 g per group, and each treatment was conducted with five biological replicates. After thorough mixing, samples were placed into polyethylene vacuum bags, sealed with a vacuum packaging machine, and fermented under dark conditions at room temperature for 45 days. After ensiling, the polyethylene vacuum bags were opened, and the corncob silage was aerobically exposed at ambient temperature for 4, 8, and 12 days. Subsequently, corncob silage samples at different aerobic exposure time points were collected using the five-point sampling method, and various parameters including mycotoxin concentrations, microbial community composition, and nutritional quality indices were determined for all collected samples.

### 2.2. Chemical Composition Analysis

After 45 days of fermentation, 200 g samples from each group were dried in the oven at 65 °C for 48 h to determine the DM content. Crude protein was determined using an N310 automatic Kjeldahl nitrogen analyzer (Guangzhou, China), and was calculated by multiplying TN by 6.25 [9]. Soluble sugars were determined using the anthrone-sulfuric acid method, while NDF and ADF contents were assessed according to the Van Soest method [10]. Crude fat was determined by the Soxhlet extraction method, and Crude Ash was determined based on the GB/T 23742-2009 standard [11,12].

### 2.3. Fermentation Quality Analysis

After 45 days of silage, 20 g samples were taken from each treatment and mixed with 180 mL of distilled water. The mixture was stored in a refrigerator at 4 °C for 24 h and then filtered through four layers of gauze and qualitative filter paper. The resulting filtrate was defined as the feed extract. The pH value of this extract was measured using a pH meter (PHBJ-260F; Shanghai INESA Scientific Instrument Co., Ltd., Shanghai, China), while the NH_3_-N content was analyzed using colorimetry based on the phenol–hypochlorite method [13]. Volatile acids were determined using a high-efficiency gas chromatograph (Agilent Technologies 7890A, Santa Clara, CA, USA), and LA content was assessed using a lactic acid kit from Nanjing Jiancheng Bioengineering Institute.

### 2.4. Determination of Mycotoxins

The concentrations of AFB1, ZEN, OT, and DON were determined in accordance with the national standard GB 13078-2017. The enzyme-linked immunosorbent assay (ELISA) kits used for detection was purchased from Shihezi Warder Biotechnology Co., Ltd., Shihezi, China, and all assays were performed strictly following the manufacturer’s instructions.

### 2.5. Microbial Community Analysis

Microbial DNA was isolated according to the Fast DNA ^®^Spin Kit for Soil (Shanghai BIOZERON Co, Ltd., Shanghai, China), with quality assessed through agarose gel electrophoresis. The primers 341F (CCTACGGGNGGCWGCAG) and 806R (GGACTACHVGGGTATCTAAT) were used to amplify the V3–V4 region of 16S rDNA. The fungal ITS region was targeted using primers TAReuk454FWD1 (5′-CCAGCASCYGCGGTAATTCC-3′) and TAReukREV3 (5′-ACTTTCGTTCTTGATYRA-3′). The PCR products were quantified using a Quanti Fluor™ ST Blue fluorescence quantification system (Promega, Madison, WI, USA) and mixed according to the sequencing volume requirements. Finally, a PE250 library was constructed, and sequencing was conducted by Shanghai Ling En Biotechnology Co. Ltd. (Shanghai, China). Sequencing was performed on the Illumina PE250 platform (Shanghai BIOZERON Co, Ltd., Shanghai, China). The obtained data were analyzed using QIIME2 software.

### 2.6. Statistical Analysis

One-way analysis of variance (ANOVA) was performed using IBM SPSS Statistics 27.0, and the relevant plots of microbial community composition were generated with Origin 2021. Multiple comparisons were conducted via Duncan’s multiple range test, where a value of *p* < 0.05 indicated a significant difference and *p* < 0.01 denoted an extremely significant difference. Results are presented as ‘mean value ± standard deviation (SD)’.

## 3. Results

### 3.1. Effects of Aerobic Exposure on the Nutritional Quality of Corncob Silage

The effects of aerobic exposure for different durations on the nutritional quality of corncob silage are presented in Table 1. With the extension of aerobic exposure time, all nutritional parameters of corncob silage were significantly affected (*p* < 0.05). Specifically, the contents of DM, CP, WSC, and ash in the T0 group were the highest, which were significantly higher than those in all other groups (*p* < 0.05). The DM content decreased to 35.19% after 4 days of aerobic exposure and further dropped to 30.31% after 8 days of exposure. In contrast, the T8 group exhibited the lowest DM and CP contents, while no significant difference in CP content was observed between the T4 and T12 groups. The WSC content decreased significantly with increasing aerobic exposure duration (*p* < 0.05). The Ash content in the T0 group was significantly higher than that in other groups (*p* < 0.05), whereas no significant differences were detected in ash content among the remaining groups. Additionally, the T0 group had the lowest NDF and ADF contents; no significant difference in NDF content was found among the other groups, while the T8 group had the highest ADF content, which was significantly higher than that in all other groups (*p* < 0.05).

### 3.2. Effects of Aerobic Exposure on the Fermentation Quality of Corncob Silage

The effects of aerobic exposure on the fermentation quality of corncob silage are summarized in Table 2. With the extension of aerobic exposure duration, the pH value of corncob silage increased significantly (*p* < 0.05). The NH_3_-N contents in the T4 and T8 groups, at 1.13% and 1.25%, were significantly lower than those in the T0 and T12 groups (*p* < 0.05), whereas the LA content after aerobic exposure was significantly lower than that in the T0 group (*p* < 0.05). Although no significant differences were detected in AA and BA contents among all groups (*p* > 0.05), the T0 group had the highest AA content (1.37 µg/mL) and the lowest BA content (0.03 µg/mL). Additionally, the PA content in the T0 group was significantly lower than that in all groups subjected to aerobic exposure (*p* < 0.05), with the T8 group showing the maximum PA content (6.67 µg/mL).

### 3.3. Mycotoxin Variations in Corncob Silage Following Aerobic Exposure

**Figure 1:**
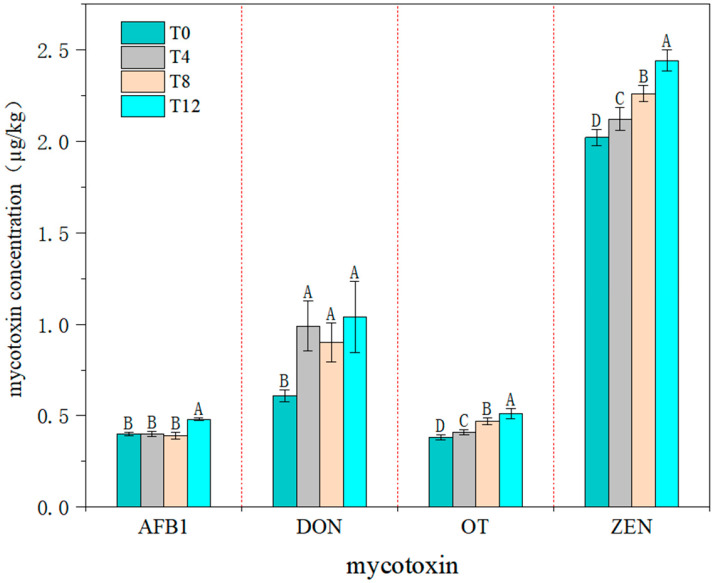
Changes in mycotoxins in corncob silage after aerobic exposure. Note: Different colors represent different days of aerobic exposure. The same letter indicates no significant difference (*p* > 0.05), while different letters indicate a significant difference (*p* < 0.05).

Variations in mycotoxin contents of corncob silage following aerobic exposure are illustrated in Figure 1. Aerobic exposure exerted a significant effect on all mycotoxin contents (*p* < 0.05). Specifically, AFB1 content in the T12 group was the highest (0.48 µg/kg), which was significantly higher than that in all other groups (*p* < 0.05), whereas no significant difference in AFB1 content was observed among the remaining groups. DON content in the T0 group was the lowest (0.61 µg/kg), and there was no significant difference in DON content among all groups subjected to aerobic exposure (*p* > 0.05). Additionally, the contents of OT and ZEN increased with the extension of aerobic exposure duration, with the T12 group exhibiting the maximum levels of these two mycotoxins. Moreover, the OT and ZEN contents in the aerobic exposure groups were significantly higher than those in the T0 group (*p* < 0.05).

### 3.4. Effects of Aerobic Exposure on the Bacterial Diversity of Corncob Silage

**Figure 2:**
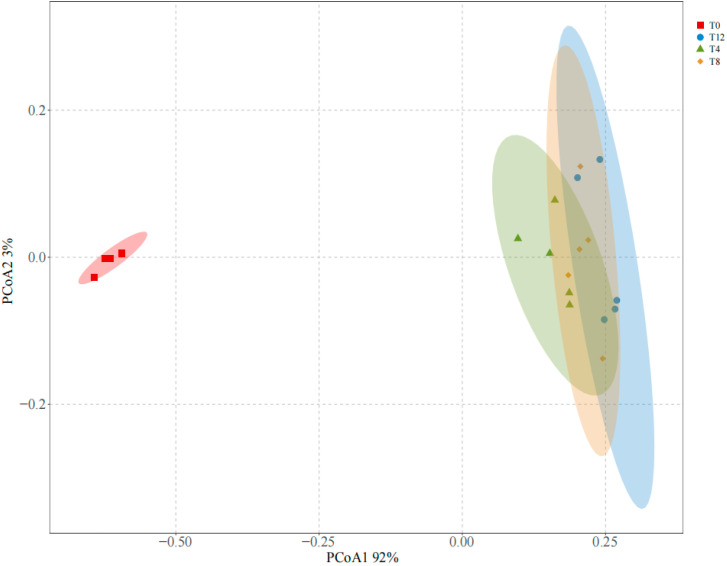
PCoA analysis of bacterial community structure in corncob silage following aerobic exposure. Note: T0 represents the corncob silage group after fermentation; T4 represents the corncob silage group after fermentation and aerobic exposure for 4 days; T8 represents the corncob silage group after fermentation and aerobic exposure for 8 days; T12 represents the corncob silage group after fermentation and aerobic exposure for 12 days; Different colors or shapes indicate different groupings.

A total of 856 amplicon sequence variants (ASVs) were obtained in this study, with the sequencing coverage of all groups exceeding 99%. As shown in Table 3, no significant difference in species richness was observed among all groups after different durations of aerobic exposure (*p* > 0.05). However, the Chao1 and ACE indices showed a slight decrease in the T0 and T4 groups, whereas the microbial richness tended to increase in the T8 and T12 groups. Furthermore, the Shannon index of the aerobic exposure groups was significantly higher than that of the T0 group, while the Simpson index was significantly lower (*p* < 0.05), indicating that aerobic exposure enhanced the microbial diversity of corncob silage. Figure 2 presents the bacterial β-diversity, from which it can be seen that there were significant differences in β-diversity between the T0 group and all groups subjected to aerobic exposure.

### 3.5. Effects of Aerobic Exposure on Bacterial Diversity at the Phylum Level in Corncob Silage

**Figure 3:**
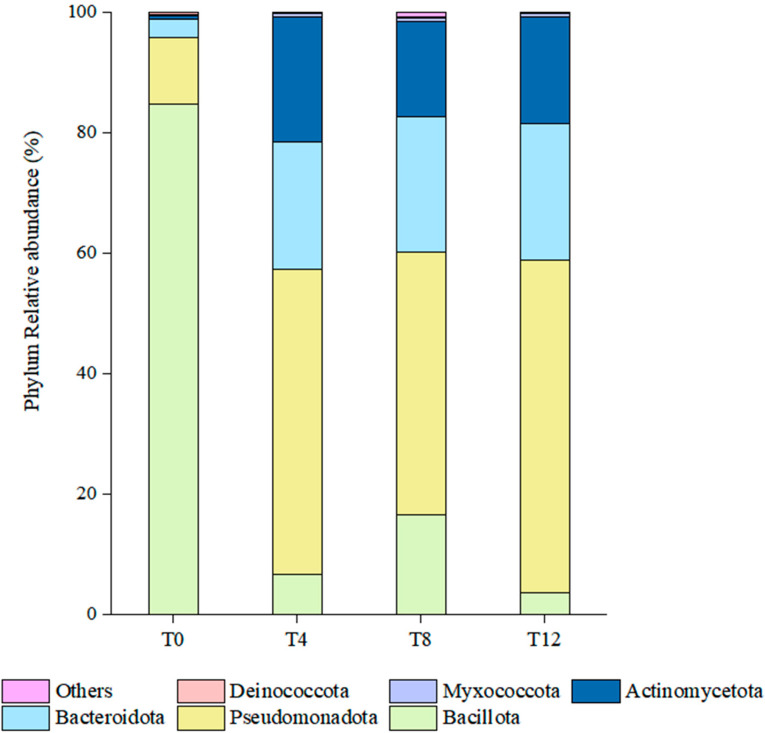
Relative abundances of bacterial phyla in corncob silage following aerobic exposure. Note: T0 represents the corncob silage group after fermentation; T4 represents the corncob silage group after fermentation and aerobic exposure for 4 days; T8 represents the corncob silage group after fermentation and aerobic exposure for 8 days; T12 represents the corncob silage group after fermentation and aerobic exposure for 12 days.

Figure 3 illustrates the bacterial diversity at the phylum level following aerobic exposure. As shown in the figure, the top three dominant phyla in the T0 group were *Bacillota*, *Pseudomonadota* and *Bacteroidota*, whereas these dominant phyla shifted to *Pseudomonadota*, *Bacteroidota* and *Actinomycetota* after aerobic exposure. With the extension of aerobic exposure duration, the dominant phylum changed from *Bacillota* to *Pseudomonadota*. Specifically, the relative abundance of *Bacillota* in the T0 group exceeded 80%, while *Pseudomonadota* accounted for the highest relative abundance (approximately 55%) in the T12 group. The relative abundance of *Bacteroidota* in the T0 group was only about 3%, whereas this value increased to around 22% in the T4, T8, and T12 groups after aerobic exposure. In addition, the relative abundance of *Actinomycetota* was even lower in the T0 group, and it ranged from 15% to 20% following aerobic exposure.

### 3.6. Effects of Aerobic Exposure on Bacterial Diversity at the Genus Level in Corncob Silage

**Figure 4:**
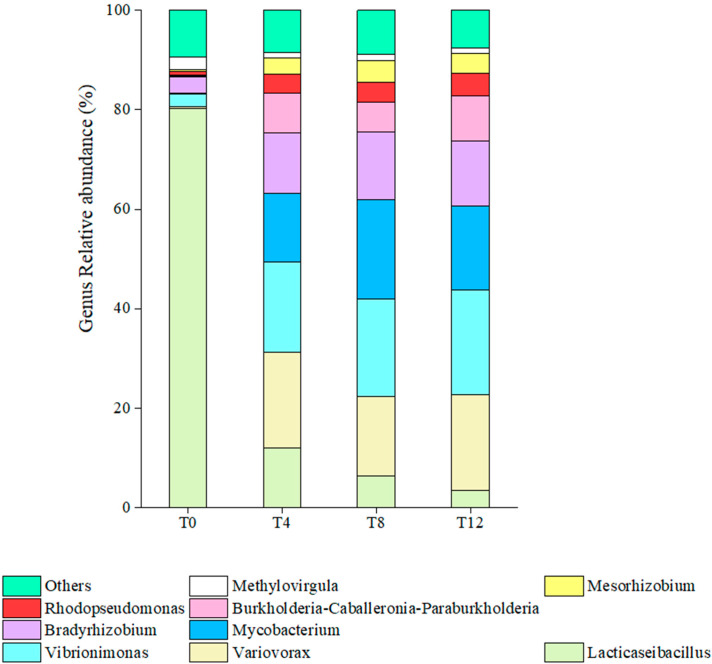
Relative abundances of bacterial genera in corncob silage following aerobic exposure. Note: T4 represents the corncob silage group after fermentation and aerobic exposure for 4 days; T8 represents the corncob silage group after fermentation and aerobic exposure for 8 days; T12 represents the corncob silage group after fermentation and aerobic exposure for 12 days.

The genus-level relative abundances of microorganisms in corncob silage after aerobic exposure are presented in Figure 4. In the T0 group, the dominant genus was *Lacticaseibacillus*, with its relative abundance reaching as high as 80%, whereas the total relative abundance of all other genera accounted for only approximately 9%. After aerobic exposure, the dominant genera shifted to *Variovorax*, *Vibrionimonas* and *Mycobacterium*, with each of these three genera contributing a relative abundance ranging from 16% to 20%. Collectively, aerobic exposure significantly altered the genus-level relative abundances of the microbial community.

### 3.7. Differential Microorganisms in Corncob Silage Before and After Aerobic Exposure

**Figure 5:**
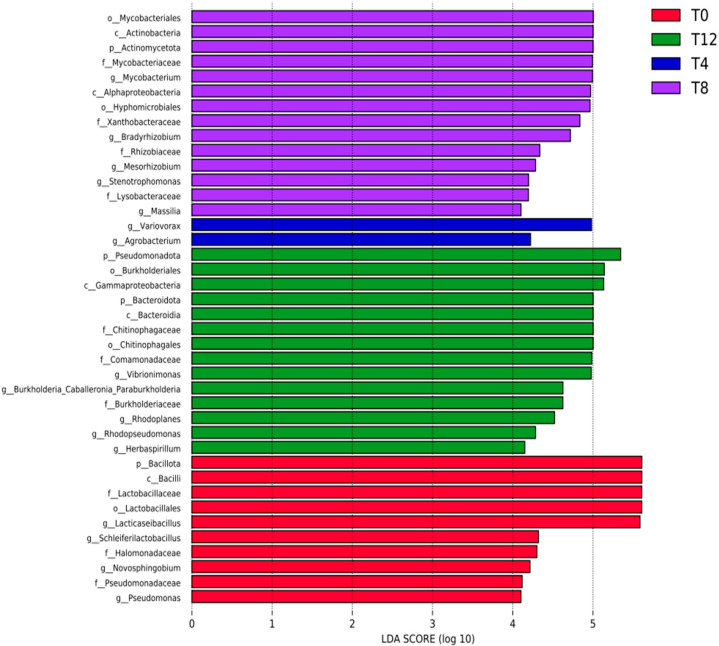
LDA histogram and evolutionary branching diagram of microorganisms in corncob silage exposed to oxygen for different numbers of days. Note: Figure 5 displays the distribution of LDA scores for different treatments. The histogram counts microbial taxa that exhibit significant effects in each group, with bar length corresponding to the magnitude of the effect size.

Linear discriminant analysis effect size (LEfSe) was employed to identify differential microorganisms across all groups. Microbial taxa with a linear discriminant analysis (LDA) score > 4 and *p*-value < 0.05 were defined as differentially abundant taxa. As shown in Figure 5, the discriminant genera in the T0 group included *g__Pseudomonas*, *g__Novosphingobium*, *g__Schleiferilactobacillus* and *g__Lacticaseibacillus*; those in the T4 group included *g__Agrobacterium* and *g__Variovorax*; those in the T8 group included *g__Stenotrophomonas*, *g__Mesorhizobium*, *g__Massillia*, *g__Bradyrhizobium* and *g__Mycobacterium*; those in the T12 group included *g__Herbaspirillum*, *g__Rhodopseudomonas*, *g__Rhodoplanes*, *g__Burkholderia_Caballeronia_Paraburkholderia* and *g__Vibrionimonas*. Collectively, these results indicated that aerobic exposure altered the composition of microbial genera in corncob silage.

### 3.8. Correlation Analysis of Differential Microorganisms with Mycotoxins and Fermentation Characteristics in Corncob Silage Pre- and Post-Aerobic Exposure

**Figure 6:**
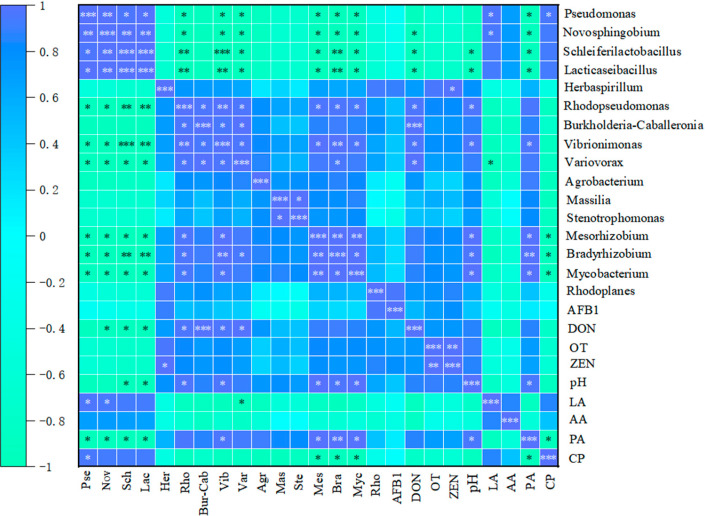
Correlation heatmap of differential genera, mycotoxins and silage fermentation parameters. Note: Different colors indicate the magnitude of correlation, with green representing positive correlation and blue denoting negative correlation. Statistical significance is denoted by asterisks: * for (*p* < 0.05), ** for (*p* < 0.01), and *** for (*p* < 0.001).

Figure 6 presents a correlation heatmap illustrating the relationships between 16 differentially abundant bacterial genera and 9 experimental indicators. As shown in the heatmap, *Pseudomonas* exhibited a positive correlation with LA and CP. *Novosphingobium* was positively correlated with LA, but negatively correlated with DON and PA. *Schleiferilactobacillus* and *Lacticaseibacillus* showed negative correlations with DON, pH, and PA. *Herbaspirillum* was positively correlated with ZEN. *Rhodopseudomonas* and *Burkholderia-Caballeronia-Paraburkholderia* both presented positive correlations with DON, and *Rhodopseudomonas* was additionally positively correlated with pH. *Vibrionimonas* was positively correlated with DON, pH, and PA. *Variovorax* was positively correlated with DON but negatively correlated with LA. Furthermore, *Mesorhizobium*, *Bradyrhizobium*, and *Mycobacterium* were positively correlated with pH and PA, while negatively correlated with CP. No statistically significant correlations were detected between *Rhodoplanes* and all the evaluated parameters in this study.

### 3.9. Effects of Aerobic Exposure on Fungal Diversity in Corncob Silage

**Figure 7:**
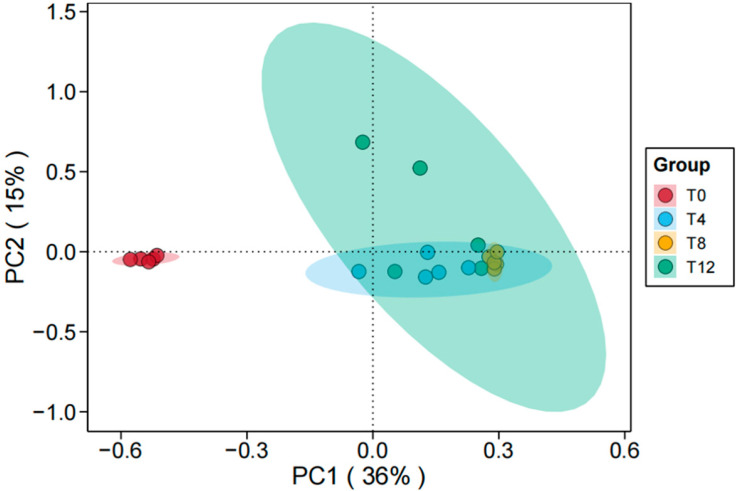
PCoA analysis of fungal community structure in corncob silage following aerobic exposure. Note: T0 represents the corncob silage group after fermentation; T4 represents the corncob silage group after fermentation and aerobic exposure for 4 days; T8 represents the corncob silage group after fermentation and aerobic exposure for 8 days; T12 represents the corncob silage group after fermentation and aerobic exposure for 12 days; Different colors indicate different groupings.

The α-diversity indices of fungi in corncob silage after 45 days of fermentation followed by aerobic exposure are presented in Table 4. The Good’s coverage values for all groups exceeded 99%, indicating sufficient sequencing depth to capture the majority of fungal taxa in the samples. A total of 24,000 ASVs were obtained across all treatments. Compared with the T0 group, the Chao1 and ACE indices were significantly higher in the aerobic exposure groups (*p* < 0.05), with the highest values observed at 4 days and 8 days of aerobic exposure, which were significantly greater than those in other groups. However, the species richness slightly decreased at 12 days of aerobic exposure. No significant differences were detected in the Shannon and Simpson indices among all groups (*p* > 0.05), though a slight reduction in fungal diversity was noted in the T8 and T12 groups. The β-diversity of fungi among different aerobic exposure periods is illustrated in Figure 7, where the T0 group was significantly separated from the aerobic exposure groups. This result confirms that aerobic exposure induced substantial changes in the fungal diversity of corncob silage.

### 3.10. Effects of Aerobic Exposure on Phylum-Level Fungal Diversity of Corncob Silage

**Figure 8:**
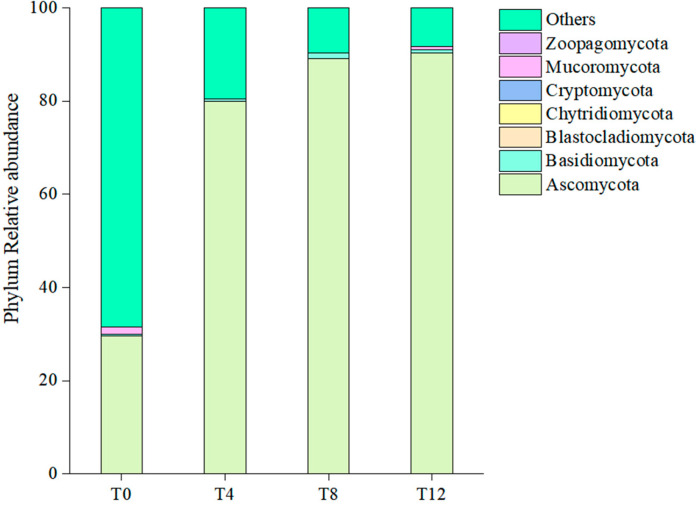
Relative abundances of fungal phyla in corncob silage following aerobic exposure. Note: T0 represents the corncob silage group after fermentation; T4 represents the corncob silage group after fermentation and aerobic exposure for 4 days; T8 represents the corncob silage group after fermentation and aerobic exposure for 8 days; T12 represents the corncob silage group after fermentation and aerobic exposure for 12 days.

Figure 8 shows the relative abundances of fungal phyla following aerobic exposure. *Ascomycota* became the dominant fungal phylum during aerobic exposure. In the T0 group, *Ascomycota* constituted approximately 30% of the community, but its relative abundance increased sharply to nearly 80% by T4 and remained stable at this high level through T12. This dominance was accompanied by a significant reduction in the “*Others*” category, which declined from 70% at T0 to approximately 10% at T4, T8, and T12.

### 3.11. Effects of Aerobic Exposure on Genus-Level Fungal Diversity of Corncob Silage

**Figure 9:**
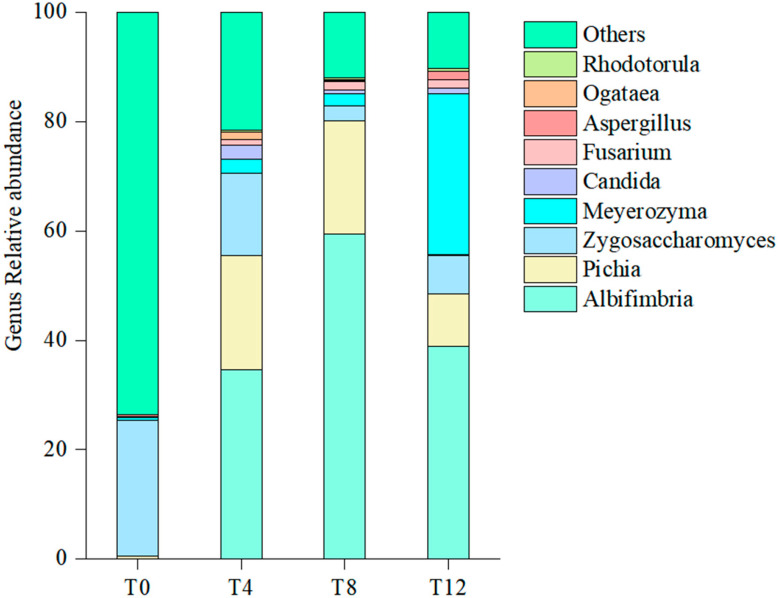
Relative abundances of fungal genera in corncob silage following aerobic exposure. Note: T0 represents the corncob silage group after fermentation; T4 represents the corncob silage group after fermentation and aerobic exposure for 4 days; T8 represents the corncob silage group after fermentation and aerobic exposure for 8 days; T12 represents the corncob silage group after fermentation and aerobic exposure for 12 days.

Figure 9 illustrates the effects of aerobic exposure on the fungal diversity of corncob silage at the genus level. At the initial stage of aerobic exposure, the dominant fungal genus in the silage community was *Zygosaccharomyces*. As the aerobic exposure period extended to 4 days and 8 days, a significant shift occurred in the dominant community structure, with *Albifimbria* and *Pichia* emerging as the core dominant genera in both groups. When aerobic exposure persisted for 12 days, the composition of dominant genera changed again, ultimately shifting to *Albifimbria* and *Meyerozyma*.

### 3.12. Differential Fungi in Corncob Silage Before and After Aerobic Exposure

**Figure 10:**
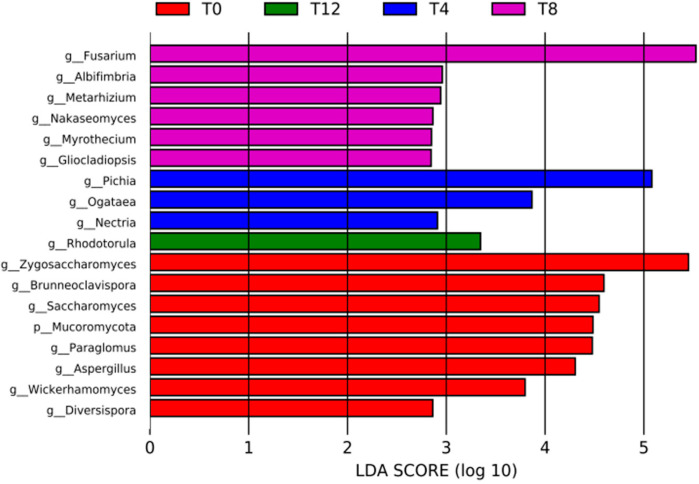
LDA histogram of microorganisms in corncob silage exposed to oxygen for different numbers of days. Note: Figure 10 displays the distribution of LDA scores for different treatments. The histogram counts microbial taxa that exhibit significant effects in each group, with bar length corresponding to the magnitude of the effect size.

LEfSe was employed to identify the differential fungal taxa across all experimental groups. Specifically, Figure 10 depicts the microbial taxa screened as differential ones with an LDA score > 4 and a *p*-value < 0.05. As illustrated in Figure 10, the differential microbial genera in the T0 group included *Zygosaccharomyces*, *Brunneoclavispora*, *Saccharomyces*, *Paraglomus*, and *Aspergillus*; in contrast, the T4 group had *Pichia* as the differential genus, while the T8 group was characterized by *Fusarium* as the differential genus.

### 3.13. Correlation Analysis Between Differential Fungi, Mycotoxins, and Fermentation Parameters in Corncob Silage Before and After Aerobic Exposure

**Figure 11:**
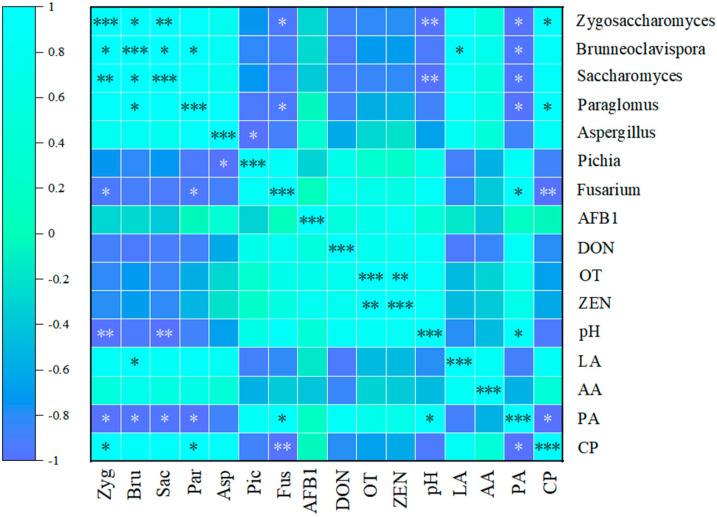
Correlation analysis between differential fungi, mycotoxins, and fermentation parameters in corncob silage before and after aerobic exposure. Note: Different colors indicate the magnitude of correlation, with blue representing positive correlation and violet denoting negative correlation. Statistical significance is denoted by asterisks: * for (*p* < 0.05), ** for (*p* < 0.01), and *** for (*p* < 0.001).

Figure 11 displays a Spearman correlation heatmap illustrating the relationships between differentially abundant fungal genera and nine experimental indicators. *Zygosaccharomyces* and *Saccharomyces* exhibited significant negative correlations with pH and PA (*p* < 0.05). *Brunneoclavispora* showed significant positive correlations with LA (*p* < 0.05), being significantly negatively correlated with PA (*p* < 0.05). *Paraglomus* was negatively correlated with PA and positively correlated with CP (*p* < 0.05). In comparison, *Fusarium* exhibited positive correlations with PA and significant negative correlations with CP (*p* < 0.05). Notably, no significant correlations were detected between differentially abundant fungal genera and mycotoxin indicators or AA.

## 4. Discussion

### 4.1. Effects of Aerobic Exposure on the Nutritional Quality of Corncob Silage

Aerobic exposure disrupts the strictly anaerobic microenvironment indispensable for successful silage fermentation, thereby inhibiting the proliferation of anaerobic taxa (with *Lactobacillus* species as the dominant group); conversely, yeasts are well recognized as the primary microbial drivers of aerobic spoilage in silage, and their vigorous growth results in the depletion of soluble carbohydrate substrates [14]. With the increase in aerobic exposure days, microbial growth consumed nutrients in corncob silage, resulting in a significant decrease in DM and WSC contents. This finding was consistent with the results reported by Zhang et al. [15]. Enhanced activities of aerobic microorganisms induced by aerobic exposure accelerated the decomposition of CP, leading to the accumulation of high levels of NH_3_-N. Meanwhile, the extensive consumption and utilization of nutrients further contributed to the reduction in DM content.

After aerobic exposure, the CP content decreased significantly. This observation was consistent with the findings of Ren et al. [16], who reported that both DM and CP contents were markedly reduced in alfalfa silage following aerobic exposure. The ingress of oxygen into silage stimulates microbial proliferation using carbohydrates and proteins as energy substrates. In addition, Chen et al. [17] proposed that elevated oxygen concentrations enhance the activity of plant proteases; moreover, most microorganisms belonging to the phylum Firmicutes possess protein-degrading capabilities. The proliferation of aerobic bacteria converts soluble sugars, proteins, and other nutrients into carbon dioxide. The gaseous loss of these nutrients consequently increases the relative proportions of NDF and ADF. However, during the aerobic exposure phase, the abundance of fiber-degrading microorganisms decreased, which alleviated the decomposition of fiber components. Wan et al. [18] demonstrated that the concentrations of NDF and ADF in whole-plant corn silage exhibited a gradual increase during the initial 0–7 days of aerobic exposure, followed by a dramatic decline after 15 days of aerobic exposure. In our study, a significant reduction in DM, WSC, and CP contents was observed in silage samples subjected to aerobic exposure compared with the T0 group, while NDF and ADF contents were significantly elevated in the aerobically exposed groups. This phenomenon could be attributed to the microbial decomposition of nutrients, and aerobic exposure inhibited the growth of fiber-degrading bacteria in the silage.

### 4.2. Effects of Aerobic Exposure on the Fermentation Quality of Corncob Silage

Aerobic exposure significantly alters key fermentation indicators of silage, thereby affecting its aerobic stability. With the increase in aerobic exposure days, the pH value increased significantly. This phenomenon was primarily attributed to the rapid consumption of LA by yeasts after the silage microenvironment shifted from anaerobic to aerobic conditions [19]; LA depletion resulted in a significant pH increase, which in turn promoted the proliferation of undesirable microorganisms. In the silage, the LA content decreased with the extension of aerobic exposure duration. LA content was negatively correlated with silage pH, meaning that a lower pH corresponded to a higher LA content [20]. Previous studies have demonstrated that aerobic exposure in silage leads to an increase in pH, elevates the risk of protein degradation, and promotes mold proliferation [21]. This is consistent with the findings of the present study, where the pH of corncob silage after aerobic exposure was significantly higher than that of the T0 group, whereas the lactic acid content was significantly lower.

NH_3_-N reflects the degree of protein and amino acid decomposition in silage. A higher NH_3_-N content indicates more extensive protein degradation, and this parameter is crucial for evaluating the aerobic stability of silage [22]. Previous studies have demonstrated that NH_3_-N content tends to increase with the progression of fermentation and the extension of aerobic exposure duration [23]. In the present study, the NH_3_-N content in the T4 and T8 groups was significantly lower than that in the T0 and T12 groups, and protein decomposition exhibited an upward trend on the 12th day of aerobic exposure. This variation was closely driven by the changes in silage bacterial communities. At T4 and T8, *Lacticaseibacillus* remained at a certain amount in the silage, effectively suppressing microbial proteolytic activity by maintaining a low pH environment [24]. Concurrently, the increased relative abundances of *Bradyrhizobium* and *Variovorax* may have driven the consumption of NH_3_-N via nitrogen assimilation [25], thereby leading to a decrease in NH_3_-N content. After 12 days of aerobic exposure, dominant bacterial groups including *Mycobacterium* accelerated the decomposition of proteins in the silage [26].

AA is the second most important organic acid in silage, and its content serves as a key indicator for evaluating nutrient degradation and diagnosing secondary fermentation. When the DM content of silage is relatively low, aerobic exposure will lead to a decrease in AA content [20]. When silage is exposed to air, AA and PA can improve the aerobic stability of the forage to a certain extent [27]. In the present study, the acetic acid content of corncob silage showed no significant difference after aerobic exposure. Furthermore, microbial inoculant application could extend the aerobic stability of the silage to a certain extent. Previous studies have indicated that adding microbial inoculants to silage increases NH_3_-N and acetic acid contents, leading to an increase in pH value. In contrast, the supplementation of lactic acid bacteria inoculants improves aerobic stability and inhibits aerobic spoilage [3].

### 4.3. Effects of Aerobic Exposure on Mycotoxins in Corncob Silage

Mycotoxins are secondary metabolites produced by fungi that pose potential hazards to human health. In natural settings, it is less likely for feed raw materials to be contaminated by a single type of mycotoxin; instead, interactive or synergistic effects often exist among different mycotoxins [28]. The most prevalent mycotoxins in corn silage include AFB1, ZEN, OT, and DON. In this study, the concentrations of these mycotoxins in Group T12 were significantly higher than those in all other experimental groups. This phenomenon could be attributed to the disruption of the anaerobic environment during ensiling, which allowed oxygen ingress and thereby facilitated the proliferation of spoilage microorganisms such as molds. Notably, mycotoxin biosynthesis is influenced by multiple factors including moisture content, relative humidity, ambient temperature, and oxygen utilization capacity, among which temperature and moisture content exert pivotal regulatory effects on mycotoxin production [16]. Qiu et al. [29] reported that the exposure of fermented feed to ambient air would trigger the proliferation of aerobic microorganisms. Mold contamination is a prevalent issue in ensiled forages during practical production. Upon aerobic exposure, the pH value of silage increases rapidly, thereby relieving the inhibitory effect on mold growth. In contrast, LAB additives have been demonstrated to mitigate the accumulation of AFB1 during aerobic exposure. In the present study, microbial inoculants were applied, and AFB1 levels exhibited a marked increase only in Group T12. This observation could be ascribed to two key factors: first, mold growth remained suppressed prior to 12 days of aerobic exposure; second, a prolonged latency period is required for molds to initiate AFB1 biosynthesis after colonization. Consistent with this finding, previous studies on corn silage have indicated that a substantial surge in AFB1 concentrations typically occurs after 14 days of aerobic exposure, and silages with poor aerobic stability are more prone to excessive aflatoxin accumulation [16].

*Fusarium* is ubiquitously present in field-grown crops, yet its relative abundance in silage samples is typically negligible, primarily due to its inability to survive under hypoxic and low pH conditions. Notably, elevated ambient temperatures can facilitate the proliferation of *Fusarium* species [30]. In our study, ZEN levels exhibited a marked upward trend with the extension of aerobic exposure duration. DON and ZEN are primarily produced by fungi of the genus *Fusarium*, and this genus was detected in all aerobic exposure groups in the present study. Consistent with previous findings, DON has been identified as the most frequently detected mycotoxin, which usually co-occurs with ZEN in corn silage samples [31]. Furthermore, the concentrations of DON and ZEN were found to be negatively correlated with the metabolic activity of LAB. Li et al. [32] reported a sharp increase in mycotoxin concentrations after 7 days of aerobic exposure in their experiment on mixed corn-soybean silage treated with the LAB strain LG608. Collectively, these observations suggest that lactic acid content and pH exert a certain inhibitory effect on mold proliferation. Oxygen ingress elevates the pH value and simultaneously facilitates mold proliferation. Huang [33] observed that the concentrations of ZEA, T-2 toxin, DON, and AFB1 increased slowly during the initial 0–3 days of aerobic exposure; as the aerobic exposure period prolonged, the rate of mycotoxin accumulation accelerated significantly. According to China’s national feed hygiene standard (GB 13078-2017), the limit of AFB1 in feed is 50 µg/kg; the maximum AFB1 content in the T12 group of the present study was only 0.48 µg/kg, and the contents of the other three mycotoxins were far below the corresponding national limit values. It is therefore hypothesized that a further extension of the aerobic exposure duration would result in mycotoxin concentrations exceeding the maximum permissible limits specified in the National Feed Standards of China.

### 4.4. Effects of Aerobic Exposure on the Bacterial Community of Corncob Silage

The Chao1 index indicates microbial richness; the higher the Shannon index, the richer the community diversity; the lower the Simpson index, the richer the community diversity; the higher the ACE index, the greater the number of microorganisms [34]. In the present study, no significant differences in Chao1 and ACE indices were observed among all groups following aerobic exposure. Specifically, these two indices exhibited a slight decreasing trend on the 4th day of aerobic exposure. Mechanistically, aerobic exposure inhibited the growth of anaerobic microorganisms involved in the silage fermentation process, leading to a reduction in the relative abundance of anaerobes. In contrast, prolonged aerobic exposure (8–12 days) facilitated the proliferation of aerobic bacteria, and the aerobic microenvironment ultimately contributed to an increase in overall microbial diversity. This finding was consistent with the results reported by Liu [35]. The growth and reproduction of harmful bacteria are the main factors leading to aerobic spoilage, and the analysis of microbial diversity showed that the diversity of bacterial species in each treatment tended to increase during aerobic exposure. A significant difference in β-diversity was observed between the T0 group and the groups after aerobic exposure.

At the phylum level, the dominant bacterial phylum in the T0 group was *Firmicutes*, which shifted to *Pseudomonadota* following aerobic exposure. This observation is consistent with the findings of Huang et al. [36], who reported that the dominant phylum switched from *Firmicutes* to *Pseudomonadota* in silages after aerobic exposure. During the aerobic exposure period, the relative abundances of *Bacteroidota* and *Actinobacteriota* gradually increased, while *Pseudomonadota* emerged as the dominant bacterial phylum. *Pseudomonadota* represent a diverse and ecologically significant group of microbes renowned for their metabolic versatility, resilience, and beneficial interactions with plants [37]. The phyla *Bacteroidota* and *Actinobacteriota* are enriched in aerobic bacteria, and the aerobic exposure environment consequently promotes the proliferation of such aerobic taxa. At the genus level, the dominant taxon in the T0 group was *Lacticaseibacillus*, with its relative abundance reaching as high as 80%. Notably, *Lacticaseibacillus* exhibits a highly complex taxonomic structure at the genus level, encompassing 170 distinct species, and this bacterial genus is typically adapted to survive under acidic conditions [38]. The dominant bacterial genera shifted to *Variovorax* and *Vibrionimonas*, both of which are classified under the phylum *Pseudomonadota*. In the anaerobic fermentation stage of silage, the relative abundance of *Pseudomonadota* was maintained at a low level. This is mainly because the rapid proliferation of lactic acid bacteria in the early stage of ensiling converts water-soluble carbohydrates into lactic acid, which forms an acidic microenvironment that inhibits the growth of *Pseudomonadota* strains sensitive to low pH [39]. The aerobic exposure stage is the key period where *Pseudomonadota* exerts a major regulatory effect on silage quality, and its relative abundance after aerobic exposure was significantly higher than that in the T0 group. Oxygen invasion after silage bag opening led to a decrease in the relative abundance of anaerobic LAB, a gradual rise in silage pH, and the destruction of the acidic anaerobic microenvironment, which provides suitable growth conditions for aerobic *Pseudomonadota*.

Correlation analysis between bacteria at the genus level and nine indicators revealed that *Pseudomonas* and *Novosphingobium* exhibited a positive correlation with LA but a negative correlation with PA, whereas the opposite trend was observed for *Schleiferilactobacillus* and *Lacticaseibacillus*. *Pseudomonas* can survive in micro-aerobic and anaerobic environments, and it plays a pivotal role in the process of meat spoilage and may be involved in the degradation of non-protein nitrogen compounds in silage [40]. *Novosphingobium* showed a positive correlation with LA and negative correlations with DON and PA. LA is the core acid-producing metabolite that maintains the low pH environment of silage; *Novosphingobium* exhibits a positive correlation with LAB, and this genus may exert a synergistic effect with LAB owing to its ability to adapt to the acidic microenvironment generated by LA accumulation [41]. In the current study, *Schleiferilactobacillus* and *Lacticaseibacillus* exhibited positive correlations with LA and negative correlations with silage pH. However, *Variovorax* showed an opposing correlative profile, with negative associations with LA and positive associations with DON. It should be emphasized that the present study only identified these potential statistical links between *Variovorax* and the aforementioned fermentation and mycotoxin indicators based on correlation analysis; no functional validation experiments were performed to deduce the specific biological mechanisms underlying these observed associations, which constitutes a key limitation of this research. Additionally, *Mesorhizobium*, *Bradyrhizobium*, and *Mycobacterium* exhibited positive correlations with pH and PA as well as negative correlations with CP. Similar to the findings for *Variovorax*, only the statistical relationships between these three genera and the above fermentation and nutritional indices were revealed in this study, without further experimental validation of their underlying biological connections. Collectively, these correlative results provide preliminary insights into the co-variation characteristics of dominant bacterial genera with key silage quality indicators, laying a foundational basis for subsequent targeted functional experiments to elucidate the ecological roles of these taxa in silage fermentation and deterioration processes.

### 4.5. Effects of Aerobic Exposure on the Fungal Community of Corncob Silage

Aerobic exposure induced an initial increase followed by a decrease in fungal community abundance, with the peak value observed at the T8 sampling point. No significant differences were detected in the Shannon and Simpson diversity indices across all groups. β-diversity analysis revealed a clear separation of microbial communities between the T0 group and aerobic exposure groups. At the phylum level, the relative abundance of *Ascomycota* in the T0 group only accounted for 30%, and it increased to 80~90% after aerobic exposure. Consistent with this finding, previous studies have reported that the application of EM inoculants also results in *Ascomycota* becoming the dominant fungal phylum in silages [42]. The dominant genus of the fungal community in corncob silage was identified as *Zygosaccharomyces*; *Zygosaccharomyces* is a well-documented osmotolerant yeast genus that can survive in the acidic and low-water-activity microenvironment of well-preserved silage. Yang et al. [43] demonstrated that the inoculation of *Zygosaccharomyces* into broad bean paste enhanced its flavor profiles. In conclusion, the succession of dominant fungal genera from *Zygosaccharomyces* to *Albifimbria/Pichia* during aerobic exposure underscores the critical role of oxygen availability in shaping the fungal community structure of corncob silage. This finding provides a theoretical basis for developing targeted strategies to mitigate aerobic deterioration, for instance, by applying antifungal additives or optimizing packaging techniques to prolong the anaerobic storage phase and suppress the growth of spoilage-related fungal genera.

Figure 11 presents the correlation heatmap between differential fungal genera and nine key indicators. Among them, *Zygosaccharomyces* and *Saccharomyces* exhibited significant negative correlations with pH and PA. In contrast, *Brunneoclavispora* displayed a significant positive correlation with LA and a significant negative correlation with PA. This correlative profile differs from that of the aforementioned yeast genera, highlighting the divergent responses of distinct fungal taxa to silage fermentation conditions. For other fermentation parameters, *Paraglomus* was negatively correlated with PA and positively correlated with CP, while *Fusarium* showed positive correlations with PA and significant negative correlations with CP. These contrasting associations between *Paraglomus* and *Fusarium* with PA and CP suggest that these genera may occupy distinct ecological niches within the silage ecosystem, though further functional validation is required to confirm this hypothesis. Notably, no significant correlations were detected between any of the differentially abundant fungal genera and mycotoxin indicators or AA.

## 5. Conclusions

This study elucidates the effects of aerobic exposure duration on the nutritional quality, mycotoxin accumulation, and microbial community structure of corncob silage. The key findings demonstrate that aerobic exposure significantly impairs silage nutritional quality, as evidenced by the reduced contents of DM, CP, WSC, and LA, and the increased contents of NDF and ADF. Concomitantly, aerobic exposure for 12 days resulted in the four mycotoxins reaching their highest levels, posing potential risks to animal health. Moreover, aerobic exposure induces profound shifts in the microbial community composition; at the phylum level, the dominant bacterial phylum shifts from *Firmicutes* to *Pseudomonadota*, the relative abundance of the fungal phylum *Ascomycota* increased from 30% in the T0 group to 80~90% in the aerobic exposure groups; at the genus level, the relative abundance of *Lacticaseibacillus* was progressively replaced by that of *Variovorax*, *Vibrionimonas*, and *Mycobacterium*; and the dominant fungal genera shifted from *Zygosaccharomyces* to *Albifimbria* and *Pichia*. These findings not only enhance our understanding of the ecological mechanisms underlying the deterioration of corncob silage during aerobic exposure but also provide critical theoretical support for the development of strategies to improve silage aerobic stability.

## Figures and Tables

**Table 1:** Changes in nutritional quality of corncob silage after aerobic exposure.

Items	T0	T4	T8	T12	*p*-Value
DM (%)	36.56 ± 0.54 A	35.19 ± 1.00 B	30.31 ± 1.06 C	31.03 ± 0.37 C	<0.001
CP (DM%)	7.83 ± 0.52 A	6.18 ± 0.43 B	5.49 ± 0.30 C	6.26 ± 0.49 B	<0.001
WSC (DM%)	2.91 ± 0.65 A	1.74 ± 0.08 B	1.57 ± 0.28 B	0.61 ± 0.29 C	<0.001
Ash (DM%)	5.25 ± 0.34 A	3.48 ± 0.37 B	3.40 ± 0.15 B	3.52 ± 0.21 B	<0.001
NDF (DM%)	62.79 ± 1.16 B	69.54 ± 1.41 A	68.39 ± 0.76 A	69.55 ± 1.78 A	<0.001
ADF (DM%)	30.41 ± 0.76 C	42.24 ± 0.70 B	44.56 ± 1.38 A	42.52 ± 0.96 B	<0.001

Note: T0 represents the corncob silage group after fermentation; T4 represents the corncob silage group after fermentation and aerobic exposure for 4 days; T8 represents the corncob silage group after fermentation and aerobic exposure for 8 days; T12 represents the corncob silage group after fermentation and aerobic exposure for 12 days; different uppercase letters in the same row indicate significant differences (*p* < 0.05). DM, dry matter; CP, crude protein; WSC, water-soluble carbohydrate; Ash, Crude Ash; NDF, neutral detergent fiber; ADF, acid detergent fiber.

**Table 2:** Changes in the fermentation quality of corncob silage after aerobic exposure.

Items	T0	T4	T8	T12	*p*-Value
pH	4.09 ± 0.01 C	4.77 ± 0.22 B	5.13 ± 0.15 A	5.15 ± 0.04 A	<0.001
NH_3_-N (TN%)	2.50 ± 0.29 A	1.13 ± 0.25 B	1.25 ± 0.26 B	2.16 ± 0.45 A	<0.001
LA (mmol/mL)	4.87 ± 0.27 A	3.17 ± 0.15 B	3.65 ± 0.54 B	3.62 ± 0.70 B	<0.001
AA (µg/mL)	1.37 ± 0.49	0.96 ± 0.56	1.28 ± 0.28	1.06 ± 0.44	0.481
PA (µg/mL)	3.21 ± 1.44 B	5.97 ± 0.93 A	6.67 ± 0.78 A	5.99 ± 1.10 A	<0.002
BA (µg/mL)	0.03 ± 0.02	0.10 ± 0.07	0.12 ± 0.08	0.16 ± 0.12	0.102

Note: T0 represents the corncob silage group after fermentation; T4 represents the corncob silage group after fermentation and aerobic exposure for 4 days; T8 represents the corncob silage group after fermentation and aerobic exposure for 8 days; T12 represents the corncob silage group after fermentation and aerobic exposure for 12 days; different uppercase letters in the same row indicate significant differences (*p* < 0.05). NH_3_-N, ammonia nitrogen; LA, lactic acid; AA, acetic acid; PA, propionic acid; BA, butyric acid.

**Table 3:** Effects of aerobic exposure on microbial diversity in corncob silage.

Item	T0	T4	T8	T12	*p*-Value
Chao1	58.60 ± 11.80	56.80 ± 2.59	60.60 ± 6.19	69.30 ± 16.18	0.286
ACE	58.60 ± 11.80	56.80 ± 2.59	60.60 ± 6.19	70.18 ± 17.23	0.288
Shannon	1.29 ± 0.19 B	2.52 ± 0.03 A	2.48 ± 0.09 A	2.43 ± 0.08 A	<0.001
Simpson	0.58 ± 0.06 A	0.12 ± 0.01 B	0.13 ± 0.01 B	0.14 ± 0.01 B	<0.001

Note: T0 represents the corncob silage group after fermentation; T4 represents the corncob silage group after fermentation and aerobic exposure for 4 days; T8 represents the corncob silage group after fermentation and aerobic exposure for 8 days; T12 represents the corncob silage group after fermentation and aerobic exposure for 12 days; different uppercase letters in the same row indicate significant differences (*p* < 0.05).

**Table 4:** Effects of aerobic exposure on fungal diversity in corncob silage.

Item	T0	T4	T8	T12	*p*-Value
Chao1	2522.36 ± 295.06 C	6865.37 ± 1173.92 A	7199.12 ± 763.69 A	5217.34 ± 862.89 B	<0.001
ACE	3003.56 ± 437.16 C	7558.61 ± 1519.07 A	7267.54 ± 1114.18 A	5641.51 ± 1214.75 B	<0.001
Shannon	5.36 ± 0.30	5.72 ± 0.24	5.37 ± 0.34	5.36 ± 0.57	0.392
Simpson	0.06 ± 0.02	0.05 ± 0.01	0.09 ± 0.04	0.09 ± 0.05	0.211

Note: T0 represents the corncob silage group after fermentation; T4 represents the corncob silage group after fermentation and aerobic exposure for 4 days; T8 represents the corncob silage group after fermentation and aerobic exposure for 8 days; T12 represents the corncob silage group after fermentation and aerobic exposure for 12 days; different uppercase letters in the same row indicate significant differences (*p* < 0.05).

## Data Availability

The original contributions presented in this study are included in the article. Further inquiries can be directed to the corresponding authors.
